# Supplementary material for: In silico analysis of local RNA secondary structure in influenza virus A, B and C finds evidence of widespread ordered stability but little evidence of significant covariation
Source: Sci Rep. 2022 Jan 10;12:310. doi: 10.1038/s41598-021-03767-x (PMC8748542; doi:10.1038/s41598-021-03767-x)
Supplement: Supplementary file 1 — Supplementary Information. [file 41598_2021_3767_MOESM1_ESM.zip › Supplemental Files/Supplemental File 3.zip!/Supplemental File 3/CM-CAll-rev/pdf/ICV_seg6_2_1.fold.R2R.sto.pdf]

ICV_seg6_2_1

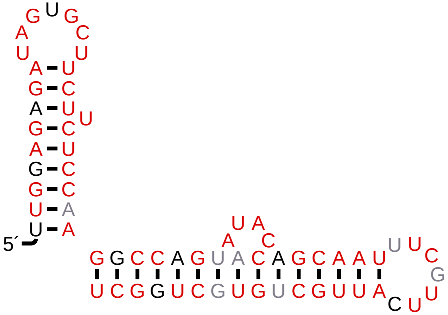

5´
GUG
A C
U U
A-U
G-C
A-U U
G-C
A-U
G-C
G-C
U-A
U-A
UA
A C
UU
GGCCAGUACAGCAAU C
G
UCGGUCGUGUCGUUA U
CU
